# Evaluating the psychometric properties of the mental health continuum-short form (MHC-SF) in Dutch adolescents

**DOI:** 10.1186/s12955-019-1221-y

**Published:** 2019-10-22

**Authors:** Chantie C. Luijten, Sofie Kuppens, Daphne van de Bongardt, Anna P. Nieboer

**Affiliations:** 1 Erasmus School of Health Policy & Management, Erasmus University Rotterdam, Rotterdam, the Netherlands; 2 Centre for Environment and Health, Department of Public Health and Primary Care, KU Leuven, Leuven, Belgium; 3 Department of Psychology, Education & Child Studies, Erasmus University Rotterdam, Rotterdam, the Netherlands

**Keywords:** Adolescents, Mental health, Well-being, Quality of life outcomes, Psychometric evaluation

## Abstract

**Background:**

Mental health is increasingly viewed as the presence of various aspects of well-being rather than just the absence of mental illness. The Mental Health Continuum-Short Form (MHC-SF) is a 14-item instrument that assesses mental health, focusing on emotional, psychological, and social well-being. The present study examined for the first time the psychometric properties of the Dutch version of the MHC-SF among adolescents, focusing on its factor structure, internal consistency, construct validity, and gender and age factorial invariance.

**Methods:**

Data were collected from a school-based sample of 1175 adolescents (53.4% girls) aged 11–17 years (M = 13.7; SD = 1.1). Participants completed an online questionnaire in the classroom during regular school hours. Statistical analyses to evaluate the factor structure, internal consistency, construct validity, and gender and age factorial invariance were performed in SPSS and R.

**Results:**

Using confirmatory factor analyses, a satisfactory-to-good fit was obtained for the three-factor model (emotional, psychological, and social well-being). The MHC-SF scores showed good internal consistency (Cronbach’s alpha = .91) and results supported convergent and divergent validity. Finally, the MHC-SF showed gender and age factorial invariance.

**Conclusion:**

The current psychometric evaluation indicates the MHC-SF is a reliable and valid instrument to assess multiple dimensions of well-being among Dutch adolescents. The instrument can be applied for research purposes and in clinical practice.

## Introduction

Although most adolescents in Western societies develop in a healthy and happy way [1], adolescence remains a period of heightened vulnerability to the onset of mental illness [2]. This vulnerability is related to the substantial physical, emotional, and social transformations that are characteristic of adolescence [3]. This includes the physical and emotional changes associated with maturation, ever-increasing academic expectations, and changing social relationships with family members and peers [1]. Indeed, epidemiological studies of mental illness among adolescents have revealed adolescent mental illness prevalence rates ranging from 10 to 20% worldwide [4], and a recent study showed that 10% of Dutch adolescents exhibited signs of mental illness, including mood disorders, anxiety, conduct problems, and substance disorders [5].

Within the literature, mental health and its relation to mental illness have been approached from different perspectives. Traditionally, mental illness has been viewed from a pathology or deficit model, which conceives mental health as the absence of mental illness [6]. However, the sole focus on treatment and prevention of mental illness has not succeeded in reducing the prevalence of mental illness in past decades [7], nor has it prevented early age of onset for mood disorders, anxiety, and substance abuse disorders [8]. A recent meta-analysis revealed that the effectiveness of mental illness treatments for children and adolescents are often modest at best and that efforts to strengthen treatments have not resulted in improved effectiveness over the past five decades [9]. Moreover, adolescents with poor well-being in the absence of mental illness appear to be equally at risk for academic and behaviour problems in school; performing no better than adolescents with a mental illness diagnosis and poor well-being [10].

Therefore, a more positive psychological approach is currently being advocated, which increases the focus on promoting and protecting well-being early in life rather than on preventing and treating deficits, like mental illness symptoms. In line with this approach, the World Health Organisation ([11] p12) defines mental health as “a state of well-being in which every individual realises his or her own potential, can cope with the normal stresses of life, can work productively and fruitfully, and is able to make a contribution to her or his community”. According to this definition, mental health is not just the absence of mental illness, but rather reflects the presence of a state of well-being, encompassing life satisfaction, positive emotions, and good functioning in one’s individual endeavours and social life.

There is a movement towards integrating symptoms with strengths and considering the balance of risks versus resources [12, 13]. For example, Keyes [14] developed a dual-continuum model, wherein mental illness and well-being are distinct, yet related, continua rather than opposite ends of a single continuum. As such, individuals with a mental illness can still experience high levels of well-being, whereas individuals without a mental illness can still experience low levels of well-being. Indeed, research has shown that an assessment that incorporates both well-being and mental illness symptoms in adolescents was a better predictor of psychosocial functioning, physical health, and school functioning than a unidimensional assessment that only considers mental illness symptoms [15, 16].

The concept of well-being itself has also evolved. Historically, well-being has been approached through either a hedonic conception, focusing on, for instance, happiness, positive affect, low negative affect, and satisfaction with life [17, 18] or a eudaimonic conception, comprising optimal functioning in one’s individual endeavours and social life, such as positive psychological functioning and human development [19–21]. The hedonic tradition is reflected in the conceptualisation of emotional well-being in terms of perceptions of avowed happiness and satisfaction with life, and the balance of positive and negative affect over a period of time. It emphasises the subjectivity of experience, including cognitive and affective evaluations of one’s life as a whole [22, 23]. The eudaimonic tradition is reflected in the conceptualisation of psychological and social well-being, with a focus on how well individuals see themselves functioning in life (e.g., purpose, personal growth, and positive relationships). Nowadays, well-being is commonly recognised as a multidimensional construct [22], encompassing emotional, psychological, and social well-being, thus, combining the hedonic and eudaimonic traditions [24].

Several instruments have been developed to assess adolescents’ well-being, most of which are rather long or measure only one or a few dimensions of well-being. The 14-item Mental Health Continuum-Short Form (MHC-SF) [25] is a relatively brief questionnaire based on the 40-item Mental Health Continuum [26]. It addresses emotional, psychological, and social dimensions of well-being, and can be used to distinguish three levels of well-being: flourishing (i.e., high levels of well-being), moderate (i.e., neither flourishing nor languishing), and languishing (i.e., absence of well-being) [14]. The MHC-SF has been shown to have good psychometric properties in both adolescents and adults within various cultural contexts, including Argentina [27], Canada [28], China [29], Egypt [30], India [31], Ireland [32], Italy [33], Korea [34], Poland [35], South Africa [25], and the USA [36, 37]. In the Netherlands, the MHC-SF has been validated for use with adults [38], but there is not yet a validated Dutch version of the MHC-SF for adolescents.

Therefore, the aim of the present study was to evaluate the psychometric properties of the Dutch version of the MHC-SF in a school-based sample of adolescents. More specifically, the objectives were: 1) to test the factor structure; 2) to examine the internal consistency; 3) to assess the construct validity, including convergent and divergent validity indices; and 4) to examine gender and age invariance of the factor structure of the MHC-SF.

In light of prior findings in adolescent samples [28–32, 34, 35] and Dutch adults [38], the factor structure of the MHC-SF was expected to confirm the three-factor structure of emotional, psychological, and social well-being. Additionally, scores of the MHC-SF subscales were hypothesised to have an adequate internal consistency and the total scores were expected to correlate positively with other measures of well-being, thereby underpinning convergent validity [29, 30, 32, 35, 36, 38]. More specifically, correlations were expected to be moderate-to-high because the dimensions of the MHC-SF subscales are similar, albeit not identical, to the validity measures [29]. In contrast, the MHC-SF total scores were expected to correlate negatively with measures of mental illness symptoms thereby supporting divergent validity and the dual-continuum model [28–30, 35, 36]. These correlations were expected to be low-to-moderate because they measure distinct, yet related continua, in line with the dual-continuum model perspective [29, 38]. Finally, the emotional, psychological, and social well-being dimensions were expected to show measurement invariance across gender and age groups [28, 29, 35].

## Methods

### Sample

Adolescents from four secondary schools located in the areas of two large cities in the Netherlands (Amsterdam and Rotterdam) participated in the present study. The sample consisted of 1175 adolescents, including 374 7th graders (31.8%), 372 8th graders (31.7%), and 429 9th graders (36.5%) between 11.0 and 17.0 years old (M = 13.7, SD = 1.1). The sample included 53.4% (*n* = 627) girls. The Dutch secondary education system encompasses different levels, including pre-vocational education (VMBO), senior general education (HAVO), and pre-university education (VWO) tracks. Most of the participants (72.8%) were enrolled in the HAVO and VWO (higher education) tracks, compared to 27.2% who were enrolled in the VMBO (lower education) track.

Almost half of the participants (49.1%) had a Dutch ethno-cultural background (i.e., adolescents and their parents were born in the Netherlands), while 42.1% had a non-Western ethno-cultural background (i.e., being born or having at least one parent born in an African, Middle Eastern, Asian, or South-American country), and 8.5% had a non-Dutch Western ethno-cultural background (being born or having at least one parent born elsewhere in Europe or in the USA, Canada, Australia, or New Zealand). Almost three-quarters of the participants (72.8%) lived with both parents in the same household.

### Procedure

Eligible schools in the Rotterdam and Amsterdam metropolitan areas were approached with an information letter and then contacted by phone and/or email one week later. Four schools provided active informed consent for their students’ participation, after which 7th, 8th, and 9th graders and their parents received online information letters describing the aims and procedure of the study. Parents had the opportunity to decline their child’s participation (passive informed consent) and adolescents were free to verbally decline participation at any the time during the study. In total, 5.8% of the parents and 0.4% of the adolescents declined participation.

Participants completed an online questionnaire in the classroom during regular school hours. Data collection was supervised by the lead researcher and several research assistants, who introduced the study and the procedure, answered questions, ensured maximum privacy, and guaranteed confidentiality of the responses. After completing the questionnaire, participants received small, non-financial incentives and a card with a list of websites to find more information about topics in the questionnaire (e.g., adolescent development, mental health) as well as the contact information of the research team to ask questions. After the data collection phase, one iPhone per school and one gift card per class (€5–€7.50 for 7th graders, €10 for 8th and 9th graders) were raffled.

The medical ethics committee of Erasmus Medical Centre, Rotterdam, the Netherlands determined that the rules stipulated in the Medical Research Involving Human Subjects Act did not apply to this study (protocol no. MEC-2018-055).

### Measures

#### MHC-SF

We used the Dutch version of the MHC-SF [14], which has previously been validated by Lamers et al. [38] in an adult sample. The original English MHC-SF [25] for adolescents is similar to the adult version, with only one adaptation to fit the adolescent population. In particular, examples of community in the item “How often did you feel that you belonged to a community?” were changed from “(like a social group, your neighbourhood, or your city)” to “(like a group of friends, at school, or in the neighbourhood)”. In the present study, we used the same adaptation for adolescents in our Dutch version.

The MHC-SF [14] consists of 14 items. Participants were instructed to think about the past month and rated the items on a 6-point scale (0 = *never*, 5 = *every day*). The items measure the degree of emotional well-being (items 1–3, e.g., “How often did you feel happy?”) in terms of satisfaction with life and the balance between positive and negative affect; psychological well-being (items 9–14, e.g., “How often did you feel good at managing the responsibilities of your daily life?”) based on Ryff’s model [20, 21]; and social well-being (items 4–8, e.g., “How often did you feel that you had something important to contribute to society?”), focusing on social acceptance, social actualisation, social contribution, social coherence, and social integration [39]. Total sum scores on the MHC-SF can range from 0 to 70, with higher scores indicating higher levels of well-being.

The MHC-SF item scores were also used to distinguish three subgroups: flourishing, moderate, and languishing. In line with previous research, participants who answered “*every day*” or “*almost every day*” at least once in the emotional well-being scale and at least 6 times across 11 items measuring social and psychological well-being were diagnosed with *flourishing*. Participants who “*never*” or “*once or twice*” experienced for at least 1 item from the emotional well-being scale and at least 6 items on the social and/or psychological well-being scales were diagnosed with *languishing*. The respondents classified neither as flourishing nor as languishing are *moderately mentally healthy* [14].

#### Other well-being measures

The participants completed four additional measures of well-being, including the Positive and Negative Affect Scale for Children (PANAS-C), the Kidscreen-27, the Social Production Function Instrument for the Level of well-being-short (SPF-ILs), and Cantril’s ladder.

##### PANAS-C

The positive affect (PA) dimension of the 10-item PANAS-C [40] was selected to measure emotional well-being, as reflected by the extent to which a person feels enthusiastic and active. The PA dimension was assessed by five items: joyful, cheerful, happy, lively, and proud. Participants rated the frequency of PA emotions on a 5-point scale (1 = *very little*, 5 = *a lot*), which were summed to yield a total score. The PA dimension has been shown to measure PA markers well among 6–18-year-olds [40]. In the present study, the Cronbach’s alpha of the PANAS-C scores was .72.

##### Kidscreen-2

The Kidscreen-27 [41, 42] is a well-being measure for young children and adolescents focused on life satisfaction, positive emotions, and feeling emotionally balanced. It consists of 27 items (e.g., “Has your life been enjoyable?”; “Have you had fun?”; “Have you felt so bad that you did not want to do anything?”). Participants were instructed to answer the questions in relation to the previous week on a 5-point Likert-type scale from 1 = *poor* to 5 = *excellent*; or from 1 = *not at all* to 5 = *extremely*; or from 1 = *never* to 5 = *always*. Four negatively formulated items were recoded according to standard procedures, after which the items were summed to yield a total score. The Kidscreen-27 has been validated with 8–18-year-olds in multiple countries, including the Netherlands [42]. The Cronbach’s alpha for the Kidscreen-27 scores in the present study was .92.

##### SPF-ILs

The SPF-ILs [43] was used to assess the extent to which adolescents’ needs for affection, behavioural confirmation, status, comfort, and stimulation are being met. This instrument was selected for validation purposes, because it measures overall well-being in terms of first-order goals that enable individuals to realise well-being. It consists of 15 items (e.g., “Do you really enjoy your activities?”; “Do you feel useful to others?”). Participants were instructed to think about the past months and rated the items on a 4-point scale (1 = *never*, 4 = *always*). Higher mean scores indicated higher levels of well-being. An adjusted version of the instrument is being used in the ongoing TRAILS (TRacking Adolescents’ Individual Lives Survey) study of adolescents and has been shown to have good reliability [44]. In the present study, the Cronbach’s alpha of the SPF-ILs scores was .86.

##### Cantril’s ladder

Cantril’s ladder [45] was used to assess current life satisfaction and reflects a general, cognitive evaluation of a person’s well-being. Respondents were asked with a single question to grade their lives on a scale from 0 to 10 with higher scores indicating higher levels of life satisfaction. Cantril’s ladder is used worldwide and has been validated among adolescents in Scotland [46].

#### Mental illness symptoms measures

##### RCADS-25

Mental illness symptoms were assessed with the Revised Child Anxiety and Depression Scale-25 (RCADS-25) [47]. The RCADS-25 is a 25-item inventory with 10 items designed to measure depressive symptoms (e.g., “Nothing is much fun anymore”) and 15 items designed to measure anxiety symptoms (e.g., “I worry about things”). The items follow a 4-point scale (0 = *never*, 3 = *always*), which are summed to yield a total score; with higher scores indicating more severe depression and anxiety. The RCADS-25 was developed for 8–18-year-old respondents and prior research supported internal consistency in a school-based and clinic-referred juvenile sample [47]. In the present study, we obtained Cronbach’s alphas of .85 for scores of the depressive symptoms subscale and .84 for the anxiety symptoms subscale.

### xStatistical analyses

Data were analysed using the Statistical Package for the Social Sciences (SPSS) version 23 [48] and R (version 3.4.3) [49] with the *lavaan* package [50]. The significance level was set at 5.0% (*p* ≤ .05).

The analyses were conducted in five steps. First**,** missing value analysis in SPSS indicated that 0.3–0.9% of the MHC-SF item scores were missing, largely as a consequence of the fact that not all participants fully completed the online questionnaire. Little’s test showed that the values were missing completely at random, χ^2^ (131) = 111.11, *p* = .895. Using the *lavaan* package, R works with full information maximum likelihood (FIML) and, thus, uses all available information.

Second, confirmatory factor analysis (CFA) was performed in R to assess the factor structure of the MHC-SF. Based on previous empirical research and theoretical considerations, three conceptual models were tested: 1) a single factor model representing general, global well-being; 2) a two-factor model comprising one latent factor representing hedonic (i.e., emotional) well-being and one factor representing eudaimonic (i.e., psychological and social) well-being; and 3) a three-factor model reflecting emotional, psychological, and social well-being. The CFA models were fitted by robust maximum likelihood (MLR) estimation because simulation studies have shown that the relative bias in parameter estimates was generally negligible, regardless of the number of ordinal categories and the shape of the observed distributions [51–53]. MLR estimation provides a test statistic that is asymptotically equivalent to the Yuan**-**Bentler T2 test statistic [54] with standard errors that are robust against violations of multivariate normality.

Satorra-Bentler (SB) χ^2^ tests were used to evaluate the absolute fit of the three models. However, because the SB χ^2^ test is considered highly conservative, potentially leading to model rejection due to very small model misspecifications in large samples [55], the following alternative indices were also used to evaluate absolute model fit: root mean square error of approximation (RMSEA) [56], comparative fit index (CFI) [57], and standardised root mean square residual (SRMR) [57]. Values of RSMEA < .06, CFI > .95, and SRMR ≤ .08 indicated a good model fit, whereas RSMEA < .08 and CFI > .90 indicated a satisfactory fit [57, 58]. As we used MLR estimation, Satorra-Bentler (SB) χ^2^ tests were used to evaluate the absolute fit of the three models.

Third, Cronbach’s alpha was used to examine the internal consistency of the MHC-SF in SPSS. A coefficient > .70 indicated good internal consistency [59]. Fourth, Pearson correlations were used to examine the construct validity of the MHC-SF in terms of convergent validity relative to alternative measures of well-being (the PA scale of the PANAS-C, Kidscreen-27, SPF-ILs, and Cantril’s ladder), as well as divergent validity relative to the RCADS-25 mental illness symptoms assessment tool. Correlations in the range of .10–.29 were considered low, those in the range of .30–.49 were considered moderate, and those ≥ .50 were considered high [60]**.**

In the fifth and final step, gender and age invariance of the best-fitting MHC-SF factor model were examined in multigroup confirmatory factor analysis. We tested configural invariance (Is the configuration of the model the same across groups?), metric/weak invariance (Are factor loadings the same across groups?), scalar/strong invariance (Are the intercepts the same across groups?), and strict invariance (Are the residual variances the same across groups?) across gender (boys vs girls) and grades (7th vs 8th vs 9th). Configural invariance was confirmed if RSMEA and SRMR were < .08 and CFA was >.95 [61]. A relative change of ≤ .010 in CFI, supplemented by a relative change of ≤ .015 in RMSEA or ≤ .030 in SRMR indicated that the null hypothesis of invariance should not be rejected [62].

## Results

### Factor structure

Table 1 presents the CFA fit indices for the three models. Model 1 and Model 2, representing a one-factor and two-factor structure, respectively, were found to have a poor absolute fit. The hypothesised three-factor model (Model 3) fitted the data significantly better than Model 1 (∆χ^2^(3) = 220.96, *p* < .001) and Model 2 (∆χ^2^(2) = 126.97, *p* < .001). All items had statisticially significant (*p* < .05) loadings on their expected factors (i.e., emotional, psychological, and social well-being), as presented in Table 2, and the fit of Model 3 was satisfactory to good.

**Table 1:** Results of the Confirmatory Factor Analyses

Model	SB χ^2^	df	*p*	RMSEA	90% CI RMSEA	CFI	SRMR
1. One factor	789.09	77	< .001	.089	.085–.094	.861	.057
2. Two factors	602.77	76	< .001	.077	.072–.082	.897	.052
3. Three factors	451.19	74	< .001	.066	.061–.071	.927	.052

*Notes. SB χ*^*2*^ Santorra-Bentler Chi Squared test, *df* degrees of freedom, *CI* confidence interval, *RMSEA* root mean square error of approximation, *CFI* comparative fit index, *SRMR* standardised root mean square residual. Criteria for interpreting model fit are: RSMEA < .08, CFI > .90, and SRMR ≤ .08

**Table 2:** Descriptive Characteristics and Factor Loadings of the MHC-SF Items

In the past month, how often did you feel…	Median	Missing (%)	Factor loadings (Model 3)		
			Emotional well-being	Psychological well-being	Social well-being
*Emotional well-being*					
1. Happy	4.00	0.3%	.78		
2. Interested in life	4.00	0.5%	.72		
3. Satisfied	4.00	0.3%	.79		
*Social well-being*					
4. That you had something important to contribute to society	3.00	0.5%			.66
5. That you belonged to a community (like a group of friends, at school or in the neighbourhood)	5.00	0.4%			.49
6. That our society is becoming a better place for people	2.00	0.6%			.78
7. That people are basically good	3.00	0.5%			.79
8. That the way our society works makes sense to you	3.00	0.5%			.71
*Psychological well-being*					
9. That you liked most parts of your personality	4.00	0.6%		.78	
10. Good at managing the responsibilities of your daily life	4.00	0.6%		.69	
11. That you had warm and trusting relationships with others	4.00	0.8%		.62	
12. That you have experiences that challenge you to grow and become a better person	3.00	0.9%		.47	
13. Confident to think or express your own ideas and opinions	4.00	0.7%		.72	
14. That your life has a sense of direction or meaning to it	4.00	0.8%		.75	

### Descriptive characteristics

Descriptive results and Pearson correlation coefficients of the MHC-SF total, subscale and item scores are presented in Table 3. Based on the three mental health categories, a majority of the participants (*n* = 638, 54.3%) experienced flourishing levels of well-being, followed by moderate levels, (*n* = 475, 40.4%), with relatively few reporting a languishing level (*n* = 62, 5.3%).

**Table 3:** Descriptive Statistics and Pearson Correlation Coefficients of the MHC-SF Subscales

Dimension	Emotional well-being	Psychological well-being	Social well-being	MHC-SF Total
M (SD)	3.88 (0.94)	3.47 (1.08)	2.93 (1.19)	3.36 (0.98)
Emotional well-being	–	−.70***	−.60***	
Psychological well-being	.63***	–	.70***	
Social well-being	.60***	.74***	–	

*Notes*: Correlations for girls are presented above the diagonal and correlations for boys below the diagonal

***p < .001

### Internal consistency

Internal consistency testing of scores of the MHC-SF total scale and subscales based on Model 3 yielded the following Cronbach’s alpha values: emotional well-being subscale, α = .80; psychological well-being subscale, α = .83; social well-being subscale, α = .81; and total MHC-SF, α = .91.

### Construct validity

The correlations of MHC-SF and the corresponding validation measures of well-being and mental illness symptoms are reported in Table 4. We observed significant positive correlations of MHC-SF scores with the PANAS-C, Kidscreen-27, SPF-ILs, and Cantril’s ladder validation measures, confirming convergent validity. Most of the correlations were high in magnitude and a few were moderate. In addition, significant negative correlations, mostly of moderate strength, were found between the MHC-SF and the RCADS-25 mental illness symptoms measure. These results supported divergent validity.

**Table 4:** Pearson Correlation Coefficients for Construct Validity

Instrument	MHC-SF well-being dimension subscale			MHC-SF total
	Emotional	Psychological	Social	
*Convergent validity*				
PA of PANAS-C	.58***	.52***	.45***	.56***
Kidscreen-27	.67***	.55***	.50***	.62***
SPF-ILs	.62***	.65***	.55***	.68***
Cantril’s ladder	.58***	.44***	.39***	.50***
*Divergent validity*				
RCADS-25	−.51***	−.46***	−.41***	−.50***
Depression	−.55***	−.48***	−.43***	−.53***
Anxiety	−.41***	−.38***	−.34***	−.41***

*** *p* < .001

*Notes*. *PA of PANAS-C* positive affect dimension of the Positive and Negative Affect Scale for Children, *SPF-ILs* Social Production Function Instrument for the Level of well-being, *RCADS-25* Revised Child Anxiety and Depression Scale-25

### Measurement invariance

The multigroup confirmatory factor analysis results are presented in Table 5. The three-factor model (Model 3) fitted the data satisfactorily across genders and grades, indicating that configural invariance was supported. Hereafter, equality constraints were imposed on all factor loadings for both gender and all grade groups. The ∆CFI, ∆RSMEA, and ∆SRMR indicated full metric invariance (< .01). Equality constraints were then imposed on all intercepts and the three difference tests also indicated full scalar invariance. Finally, equality constraints were imposed on all residual variances, with the ∆CFI, ∆RSMEA, and ∆SRMR supporting full strict invariance.

**Table 5:** Measurement Invariance across Gender and Grades (Model 3)

Model	SB χ^2^	df	RMSEA	∆RMSEA	CFI	∆CFI	SRMR	∆SRMR
Gender invariance								
1. Configural	735.84	148	.083	–	.919	–	.053	–
2. Metric	750.37	159	.080	.003	.919	.000	.055	.002
3. Scalar	806.59	170	.080	.000	.913	.006	.059	.004
4. Strict	845.14	184	.079	.001	.909	.004	.060	.001
Grade invariance								
1. Configural	806.67	222	.083	–	.921	–	.055	–
2. Metric	826.44	244	.079	.004	.921	.000	.059	.004
3. Scalar	872.13	266	.077	.002	.918	.003	.061	.002
4. Strict	956.77	294	.076	.001	.910	.008	.064	.003

*Notes. SB χ*^*2*^ Santorra-Bentler Chi Squared test, *df* degrees of freedom, *CI* confidence interval, *RMSEA* root mean square error of approximation, *CFI* comparative fit index, *SRMR* standardised root mean square residual. Criteria for interpreting model fit are: RSMEA < .08, CFI > .90, and SRMR ≤ .08

## Discussion

To evaluate the psychometric properties of the Dutch version of the MHC-SF in a school-based sample of adolescents, we tested the factor structure and invariance and assessed internal consistency and construct validity of the MHC-SF. All items loaded significantly on their expected factors, consistent with prior research in adolescents [28–32, 34, 35] and in Dutch adults [38]. CFA-analyses confirmed the three-factor structure of the MHC-SF. The three-factor model was the best-fitting model to these data, suggesting that the items measuring emotional, psychological, and social well-being are reflections of three distinct but correlated latent factors. The goodness of fit of the three-factor model was satisfactory to good and comparable to, sometimes even better than, results from prior studies in adolescent and adult samples [25, 27–37].

We observed good internal consistency for the MHC-SF and, as expected, moderate-to-high associations with well-being validation measures—namely PA of the PANAS-C, Kidscreen-27, SPF-ILs, and Cantril’s ladder—supporting convergent validity. Some measures, such as the Kidscreen-27 and SPF-ILs, cover a broader conceptualisation of well-being than others like the PANAS-C and Cantril’s ladder. The well-being validation measure SPF-Ils, for example, correlated strongest with total MHC-SF score, an assessment of overall well-being. This result is not surprising given that the SPF-ILs is used as an overall well-being measure encompassing social and physical well-being subdimensions. On the other hand, the PANAS-C and Cantril’s ladder instruments are more specific measures of emotional well-being and life satisfaction and indeed correlated strongest with the MHC-SF emotional well-being subscale. Thus, the MHC-SF is a reliable and valid instrument to assess well-being of adolescents.

The mostly moderate associations of the MHC-SF with mental illness symptoms (i.e., anxiety and depression symptoms) supported divergent validity. Previous studies involving adolescents and Dutch adults have also shown divergent validity of the MHC-SF vis-à-vis correlations with anxiety and depression symptoms [25, 31, 33–35, 38]. Consistent with the dual-continuum model, in which mental health and mental illness are conceptualised as two distinct, yet related continua, our results showed correlations between mental health and mental illness symptoms measures, with some divergence. The correlations were weaker compared to the correlations with the well-being validation measures, but still significant. This indicates that the absence of mental illness does not necessarily imply the presence of well-being and emphasises the importance of a positive psychological approach in the assessment of mental health.

Finally, full strict invariance was observed by gender and grade. The MHC-SF structure, factor loadings, intercepts, and residual variances were the same in boys and girls, and also the same across different age groups, in line with our expectations based on recent studies [28, 29, 33, 35]. These findings indicate that the MHC-SF measures well-being with the same level of accuracy in boys and girls and with the same level of accuracy across different age groups, which supports broad usage of the MHC-SF to measure well-being in adolescent populations.

### Limitations and future research directions

Although this study was strengthened by the use of a large sample of Dutch adolescents (*N* = 1175), the national generalisability of our results may have been compromised by our use of a school-based sample. To examine the representativeness of this sample, a comparison was made to the Health Behaviour in School-age Children (HBSC) study, where the sample is considered to be representative of the general Dutch adolescent population [63]. This comparison showed that our sample may be considered representative with respect to gender, age, and household characteristic variances. However, it differed from the HBSC study regarding ethnicity and education level in that our cohort had over-representations of participants with a non-Western ethno-cultural background (42% vs. 17%) and with a high education level (73% vs. 53%). These differences may be explained by the fact that the four involved schools were located in two relatively diverse metropolitan areas that tend to have more adolescents with a non-Western ethno-cultural background than other, less urban areas in the Netherlands. In addition, the involved schools consisted of more adolescents with a high educational level. The involvement of more schools with diverse educational levels is, therefore, recommended in future research.

Ethnicity and educational level are important determinants of health and well-being [1] and, therefore, the over-representations of a non-Western ethno-cultural background and high education level may have biased our results. However, in accordance with Dutch adolescents being among the happiest and most satisfied with their lives around the world [63], the percentage of flourishing adolescents (54.3%) in this study was much higher than the percentages reported for other adolescent samples (23.4% in Egypt [30]; 11.7% in Korea [34]).

Furthermore, it should be noted that our data were hierarchical in nature with students nested in grades and schools. When we evaluated the necessity of using multilevel models, we found some variance at level of grade, but not of school. This variance was, however, fairly small and did not affect our conclusions (data available upon request). Consequently, we performed our analyses without accounting for the nested structure.

It is recommended that, in addition to emotional, psychological, and social well-being, a dimension of physical well-being be included in future research to assess adolescents’ overall well-being. Keyes’ dual-continuum model and the MHC-SF focus on emotional, psychological, and social well-being as positive mental health. However, there is consensus that mental and physical health are deeply interdependent [64]. Moreover, the MHC-SF includes only positive items to measure well-being even though the dual-continuum model proposes that well-being can be achieved even when there are negativities such as mental illness or feelings of negative affect like sadness. Negative aspects of well-being are likely to be relevant for social and psychological well-being, especially with respect to troubled social relationships or social dysfunction. Finally, future studies are needed to validate the MHC-SF in clinical adolescent populations and to perform cross-country comparisons of measurement invariance.

## Conclusion

This is the first study to evaluate psychometric properties of the Dutch version of the MHC-SF in an adolescent sample. The results indicated that the MHC-SF is a valid and reliable instrument that can be administered to assess different dimensions of well-being in adolescents across mental health, education, and health policy contexts. The brevity of the MHC-SF (14 items) and its cross-contextual utility make it highly suitable for scientific and epidemiological monitoring of adolescent mental health and well-being. It may also provide a useful tool that can be used across interventions and prevention strategies because of the importance to promote and protect well-being in addition to reducing mental illness symptoms. The ability to distinguish among flourishing, moderate, and languishing adolescents creates the possibility to manage multiple dimensions of well-being to ensure more complete mental health and to choose and implement interventions that are informed by an individual’s position along both the mental illness and well-being dimensions. Ultimately, the ability to properly assess the complexity and multidimensionality of well-being may lead to more effective improvements in adolescent mental health and related developmental outcomes. The present findings enable future comparisons between adolescent samples across countries and contribute to the extensive use of the MHC-SF in research and clinical practice.

## Data Availability

The dataset used and analysed during the current study is available from the corresponding author on reasonable request.

## Abbreviations

CFA: Confirmatory factor analysis
CFI: Comparative fit index
FIML: Full information maximum likelihood
HAVO: Senior general education track
HBSC: Health Behaviour in School-age Children
MHC-SF: Mental Health Continuum-Short Form
MLR: Robust maximum likelihood method
PA: Positive affect
PANAS-C: Positive and Negative Affect Scale for Children
RCADS-25: Revised Child Anxiety and Depression Scale-25
RSMEA: Root mean square error of approximation
SB: Satorra-Bentler
SPF-ILs: Social Production Function Instrument for the Level of well-being scale
SRMR: Standardised root mean square residual
TRAILS: TRacking Adolescents’ Individual Lives Survey
VMBO: Pre-vocational education track
VWO: Pre-university education track
